# Multidimensional insights into exosomes in hepatocellular carcinoma: from genesis to clinical application

**DOI:** 10.3389/fimmu.2025.1628573

**Published:** 2025-08-13

**Authors:** Xiang Zhang, Cheng Zhang, Zheng Zhang, Xuan Zhang

**Affiliations:** ^1^ School of Anesthesiology, Wannan Medical College, Wuhu, Anhui, China; ^2^ School of Clinical Medicine, Wannan Medical College, Wuhu, Anhui, China; ^3^ School of Clinical Medicine, Bengbu Medical University, Bengbu, Anhui, China; ^4^ Department of General Surgery, The Affiliated Hospital of Xuzhou Medical University, Xuzhou, Jiangsu, China

**Keywords:** hepatocellular carcinoma (HCC), exosomes, tumor microenvironment (TME), drug tolerance, metastasis, targeted therapy

## Abstract

Hepatocellular carcinoma (HCC) is a highly aggressive malignancy, whose progression is intimately linked to the complex dynamics of the tumor microenvironment (TME). Exosomes, once considered mere cellular waste, have emerged as pivotal mediators of intercellular communication within the TME, actively participating in the multistep development of HCC. These nanoscale vesicles play crucial roles in the initiation of precancerous lesions and, by transporting drug resistance-related molecules such as proteins and non-coding RNAs, facilitate the acquisition of resistance to chemotherapy and targeted therapies by tumor cells. Moreover, exosomes contribute to the establishment of pre-metastatic niches by remodeling distant organ microenvironments—inducing hypoxia, metabolic reprogramming, and angiogenesis—which collectively create favorable conditions for tumor cell colonization. They also modulate immune responses by inducing T-cell exhaustion, promoting macrophage polarization, and disrupting normal stromal cell functions, thereby constructing an immunosuppressive microenvironment that enables tumor immune evasion. Given their inherent biocompatibility and targeting capabilities, engineered exosomes have shown promise in cancer therapy, serving as carriers for therapeutic molecules and enabling precise drug delivery through surface modifications. Despite significant advancements, challenges remain in elucidating the *in vivo* regulatory mechanisms of exosomes, standardizing their isolation and purification processes, and evaluating their clinical efficacy. This review examines the multifaceted roles of exosomes in HCC, aiming to bridge mechanistic insights with precision diagnostics and pave new avenues for liver cancer treatment.

## 1 Introduction

HCC ranks as the 6th most prevalent cancer and 4th leading cause of cancer-related mortality. It constitutes approximately 90% of primary liver cancer cases and is anticipated to exceed one million new diagnoses annually by 2025 (1–3). Despite improved treatments, HCC prognosis remains grim with a 5-year survival rate <20%, worsened by rising risk factors like viral hepatitis, non-alcoholic fatty liver disease (NAFLD), and alcoholic liver disease (ALD)—all closely linked to HCC pathogenesis (4, 5). These chronic liver diseases not only heighten the risk of HCC but also propel its progression through multiple mechanisms. Current HCC treatments include surgical resection, liver transplantation, chemotherapy, and targeted therapies. Systemic therapy has evolved from single-agent targeted therapies (e.g., sorafenib and lenvatinib) to combinations of immune checkpoint inhibitors and targeted therapies (e.g., atezolizumab combined with bevacizumab). However, tumor heterogeneity and drug resistance often diminish efficacy, with few patients gaining long-term benefits and overall prognosis remaining poor. Its growing incidence elevates HCC’s lethal threat (6–8). The tumor microenvironment (TME) comprises immune cells, stromal cells, extracellular matrix components, and a repertoire of cytokines. Intercellular signaling and dynamic interactions within this niche intricately modulate tumor growth, invasive potential, and metastatic dissemination (9). Exosomes have recently attracted considerable attention as key mediators of intercellular communication (10). These 30–150 nm membrane-bound vesicles are secreted by all cell types, including both prokaryotic and eukaryotic cells, under both normal and disease conditions. Initially regarded as simple byproducts of metabolism, exosomes are now recognized for their crucial role in intercellular signaling (11–13), They can carry a range of biologically active molecules, such as proteins, lipids, mRNAs, and miRNAs, all of which can significantly influence the behavior of target cells.In HCC, exosomes are implicated in immunosuppression, tumor drug resistance, formation of pre-metastatic niches, and remodeling of the tumor microenvironment (14, 15). Tumor-derived exosomes carry tumor-specific molecules, supporting their potential as novel early detection tools (16). Additionally, their low toxicity, low immunogenicity, and engineerable nature enable drug loading and targeted delivery, offering promise for combination therapies (17, 18).

By systematically summarizing and analyzing existing studies, this review aims to offer new perspectives and insights for the diagnosis and treatment of HCC, thereby promoting further research in this field.

## 2 Exosome promotes the development of precancerous liver disease

In recent years, growing research focus on exosomes’ functions and molecular mechanisms in cancer pathogenesis. As key mediators of intercellular signaling, exosome regulate a spectrum of pathobiological behaviors (19). They play pivotal roles in the progression of precancerous liver conditions—including viral hepatitis alcoholic liver disease (ALD), and fatty liver disease,—by driving HCC development, metastasis, and hepatic fibrosis (4, 20–22).

In viral hepatitis, exosomes are integral to pathophysiology, directly promoting viral replication and transmission (23). For instance, the hepatitis A virus (HAV) is released from the host cell membrane to produce eHAV, which is transmissible, circulates in infected individuals, and resists chloroform (24). Host proteins involved in intracellular trafficking (e.g., VPS4B, ALIX) are required for eHAV biogenesis. Additionally, HCV replication may depend on the host endosomal sorting complex (ESCRT), shedding new light on exosomes’ role in viral spread (25). Exosomes produced from HCV-infected cells could also transfer HCV to uninfected human HCC Huh7.5.1 cells and create a helpful infection (26). Interestingly, differentiation-antagonistic non-protein-coding RNA (lncRNA) levels were elevated following HCV infection, and HCC linked to the hepatitis C virus showed higher expression of this lncRNA (27). Exosomes further contribute to hepatic fibrosis—a key pathological response to severe liver injury. For instance, miR-222-rich exosomes from HBV-infected LO2 hepatocytes activate mouse hepatic stellate cells (LX-2) via the transferrin receptor (TFRC), accelerating fibrosis (28).

Regarding ALD, ethanol significantly increases extracellular Vesicles (EVs) secretion by HepG2 cells, closely associated with caspase-3 activation (29). Exosomal miR-155 drives inflammation and fibrosis by regulating autophagy and lysosomal dysfunction, and is critical in ALD—highlighting potential for early diagnosis and treatment (30). Alcohol-fed mice exhibit elevated circulating EVs; microarray analyses reveal dysregulated inflammatory miRNAs (e.g., miRNA-192, -122, -30a) in serum compared to controls, with their upregulation linked to progressive liver inflammation and fibrosis (31).

Exosomes also play critical roles in nonalcoholic fatty liver disease (NAFLD). Under high-fat, fructose, and cholesterol diets, activated IRE1A promotes hepatocyte release of ceramide-rich inflammatory EVs, which recruit monocyte-derived macrophages to the liver, inducing inflammation and injury (32). Additionally, Lee et al. found that human HCC cell lines treated with palmitic acid secreted significantly more exosomes, which notably upregulated fibrosis-related genes and promoted fibrosis development when taken up by hepatic stellate cells (HSC) (33). Further studies revealed that after incubation with palmitate or lysophosphatidylcholine, primary hepatocytes and human HCC Huh7 cells showed increased EV release. In mouse macrophages, this release may trigger an inflammatory response by causing the production of interleukin 1β (IL-1β) and interleukin 6 (IL-6) (34). In addition, the development of hepatic and systemic insulin resistance (IR), as well as steatosis, is linked to adipocyte-derived EVs in NAFLD. Hepatocytes generate EVs in response to lipotoxicity, and these EVs aid in the development of fibrosis by stimulating macrophages and hepatic stellate cells (HSCs) (35). Understanding these processes provides a complete framework for unraveling the pathophysiology of precancerous liver disease, making it possible to discover potential therapeutic targets and develop novel treatment options.

## 3 Exosome-mediated mechanisms of multi-drug resistance formation

Tumor drug resistance is a complex, multi - dimensional process involving multiple mechanisms: exosomes transmit drug efflux proteins (e.g., P - glycoprotein [P - gp]) between cells, which not only enhances the resistance of tumor cells but also spreads this trait to drug - sensitive cells (35). Another important molecular mechanism behind drug resistance is the metabolism of chemotherapeutic drugs by the enzyme cytochrome P450 (CYP) (36). Also, an important advancement in studying drug resistance in cancers has been facilitated by the exosome-mediated non-coding RNA (ncRNA) regulatory network.

### 3.1 Drug efflux proteins -mediated resistance transmission

P-glycoprotein (P-gp) can be transferred between cells via two pathways independent of drug selection pressure. P-gp can be transferred remotely by the release of particles, which can be effectively detected within 4 h of release. In contrast, contact transfer is mediated by tunneling nanotubes that form “bridges” between neighboring cells (37). For example, paclitaxel-resistant A2780/PTX ovarian cancer cells secrete P-gp-carrying microvesicles (MVs). These microvesicles transferred P-gp into chemotherapy-sensitive A2780/WT cells in a time- and dose-dependent manner, significantly enhancing the latter’s resistance (38). Moreover, the ABCB1 gene encodes P-glycoprotein (P-gp). Upon exposure to chemotherapeutics, drug-resistant cells increase release of ABCB1-carrying EVs, mediated by Rab8B.Furthermore, in drug-sensitive tumor cells, Rab5 downregulation accelerated the recycling of these exosomes to the plasma membrane. This process makes it easier for ABCB1 to move between cells, which allows normally susceptible cells to quickly develop resistance. This resistance is temporary, though, and mainly helps cells avoid the cytotoxic effects of chemotherapy medications (39).

### 3.2 Enzyme cytochrome -mediated resistance transmission

Beyond drug efflux, cytochrome P450 (CYP) enzyme-mediated metabolism of chemotherapeutics has recently been identified as a key biological driver of treatment resistance in cancer. Chemotherapeutic medicines can be reduced in cytotoxicity by being converted into inactive or low-toxicity metabolites by the CYP enzyme system, which is essential for drug metabolism (40). These exosomes deliver enzymes to target cells via membrane fusion or internalization, further enhancing drug metabolism (41).

### 3.3 Non-coding RNA-mediated resistance transmission

**Figure 1:**
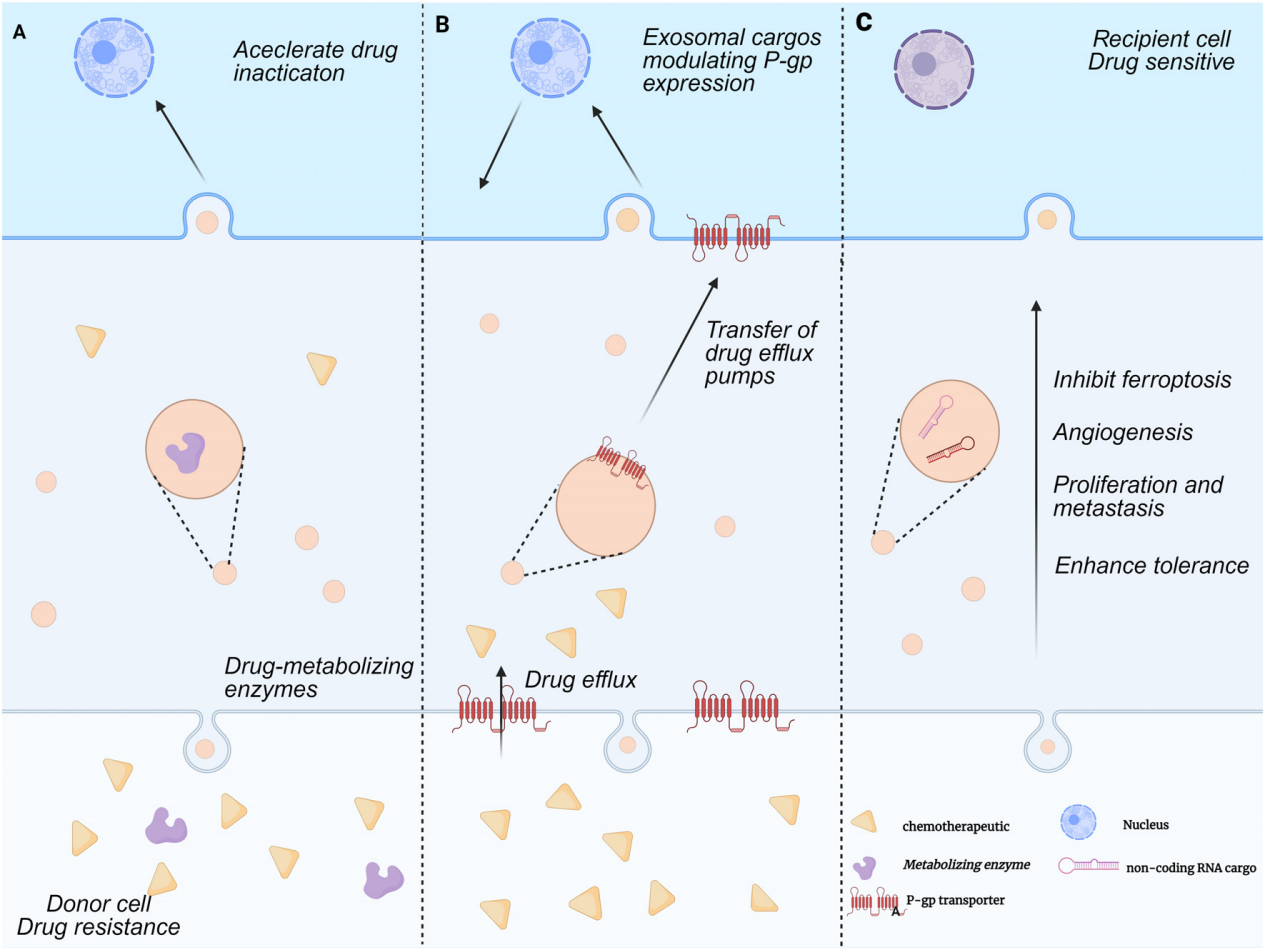
Mechanisms of exosome-mediated multi-drug resistance formation. This figure illustrates the exosome-mediated tumor drug resistance process between donor and recipient cells, which includes three parts:**(A)** Accelerated drug inactivation:Donor cells with drug metabolizing enzymes release exosomes carrying these enzyme-related substances. These exosomes act on recipient cells to accelerate drug inactivation, conferring drug resistance. **(B)** Regulation of P-glycoprotein expression and drug efflux pump transfer:Donor cell-derived exosomes carry cargoes that regulate P-gp expression and transfer drug efflux pumps to recipient cells. Recipient cells then expel chemotherapeutic drugs, creating drug resistance. **(C)** Promotion of tumor progression:Donor cell-derived exosomes carry non-coding RNA and other substances. These exosomes promote cell proliferation, inhibit ferroptosis, induce angiogenesis, and enhance cellular tolerance to drug sensitization in recipient cells, thereby enhancing tumor resistance to chemotherapeutic drugs.

Chemoresistance in HCC is a complex multifactorial process, with the exosome-mediated non-coding RNA (ncRNA) regulatory network emerging as a key breakthrough in mechanistic studies in recent years. Exosomes remodel the tumor microenvironment and activate drug resistance-associated signaling pathways by delivering ncRNA molecules (e.g., miRNAs, lncRNAs, circRNAs) (42). To regulate post-transcriptional alterations in gene expression, exosomes generated from HCC cells deliver these non-coding RNAs to target cells via endocrine or paracrine pathways. Exosomes, for example, have the ability to move circDCAF8 from HCC cells that are resistant to regorafenib to those that are susceptible to it. Regorafenib-sensitive cells can develop a drug-resistant phenotype through this translocation process (43). Similarly, circRNA-SORE is translocated by exosomes to propagate sorafenib resistance in HCC cells (44). Further studies indicate that exosomes from sorafenib-resistant HCC cells are enriched with circUPF2, which enhances sorafenib resistance by promoting SLC7A11 expression and inhibiting ferroptosis.circUPF2 enhances sorafenib resistance by stabilizing the circUPF2-IGF2BP2-SLC7A11 ternary complex, which stabilizes SLC7A11 mRNA, promotes systemic Xc^-^ function, and reduces ferroptosis sensitivity, thereby inducing drug resistance (45). Exosomal circCCAR1, when taken up by CD8+ T cells, contributes to CD8+ T cell dysfunction by stabilizing PD-1 proteins, which in turn leads to resistance to PD-1 immunotherapy (46). Notably, exosomes can enhance cellular autophagy, thereby increasing tumor cell tolerance. For example, circTGFBR2, which is transported to HCC cells by exosomes, acts as a competitive endogenous RNA by binding miR-205-5p, which increases autophagy and ATG5 expression, making HCC cells more resistant to starvation (47) (**Figure 1**).

## 4 Exosomes as Architects of the Pre-Metastatic Niche

### 4.1 Hypoxia, angiogenesis, and metabolic reprogramming: exosomal regulation of the pre-metastatic niche in hepatocellular carcinoma

Primary tumors induce adaptive alterations in the microenvironment of distant organs prior to metastasis, creating favorable conditions for tumor colonization and growth—a process referred to as pre-metastatic niche (PMN) formation (48). By modifying hypoxia, promoting angiogenesis, and reprogramming tumor metabolism, exosomes help establish the PMN, thereby facilitating metastasis by preparing secondary organs to support circulating tumor cells and promote their survival and proliferation (49).

Hypoxia is a key component of the microenvironment in solid tumors, particularly hepatocellular carcinoma (HCC). Both hypoxia and the consequent oxidative stress play important roles in the progression and treatment of HCC. Aside from increasing tumor cell invasiveness and stem-like qualities, hypoxic circumstances also drive cancer cells to create more cytokines, non-coding RNAs, and exosomes (50, 51). For instance, hypoxia-exposed HCC cells package miR-1290 into exosomes, which facilitates M2 macrophage polarization by downregulating Akt2 and upregulating PD-L1. This phenotypic shift in macrophages induces CD8+ T cell apoptosis and promotes epithelial-mesenchymal transition (EMT), thereby enhancing HCC cell migration (52). Likewise, miR-4508 expression is elevated in tumor-derived exosomes (TDEs) under hypoxic stress. This miRNA targets the 3′ untranslated region (3′UTR) of regulatory factor X1 (RFX1), leading to its downregulation. Reduced RFX1 levels activate fibroblasts and promote pre-metastatic niche (PMN) formation via the IL17A–p38 MAPK–NF-κB signaling cascade. These activated fibroblasts subsequently aid in the recruitment and expansion of myeloid-derived suppressor cells (MDSCs). Consequently, the lung PMN established via fibroblast activation facilitates metastatic spread of primary tumors to the lungs by establishing a favorable tumor colonization environment (53).

HCC frequently undergoes metabolic reprogramming, characterized by enhanced aerobic glycolysis—a phenomenon termed the Warburg effect.This metabolic pattern effectively generates energy and vital macromolecules for cell growth, thereby driving swift tumor expansion and proliferation. It was discovered that tumor-associated macrophage (TAM)-derived exosomes containing lncMMPA play important roles in macrophage polarization, HCC glycolytic pathway activation, and cell proliferation. In particular, TAM-derived lncMMPA promotes M2-type macrophage polarization. Elevated lncMMPA levels in HCC promote cell proliferation and aerobic glycolysis by stabilizing aldehyde dehydrogenase 1A3 (ALDH1A3) (54). In HCC, tumor exosomes carrying fatty acids—particularly palmitic acid—stimulate hepatic macrophages to secrete tumor necrosis factor (TNF), leading to a pro-inflammatory microenvironment that impairs fatty acid metabolism and oxidative phosphorylation, thereby promoting fatty liver progression (55). In the pre-metastatic niche, cancer cells secrete miR-122-rich vesicles to restrict glucose uptake by non-tumor cells (56). Interestingly, it has been shown that breast cancer (BC) cells inhibit pyruvate kinase (PKM) in pancreatic islet β-cells by secreting miR-122-rich extracellular vesicles, which in turn reduces insulin secretion and leads to dysregulation of glucose homeostasis. In addition to encouraging tumor growth, this mechanism raises the risk of type 2 diabetes in BC patients (57).

The advancement and dissemination of HCC are significantly contingent upon angiogenesis. Sustained multiplication of tumor cells is dependent on an appropriate supply of nutrients and oxygen, and the neovascular network formed by angiogenesis is vital for meeting this demand. Exosomes secreted by HCC cells is crucial to pathological angiogenesis by delivering various pro-angiogenic molecules (58). Golgi protein 73 (GP73), which is significantly upregulated in HCC, stimulates the production and secretion of vascular endothelial growth factor A (VEGFA) and promotes angiogenesis in the tumor microenvironment (59). According to clinical studies, individuals with HCC and high miR-3174 expression have a worse prognosis. Under hypoxia, miR-3174 is increasingly packaged into exosomes, facilitating angiogenesis and metastasis in HCC by suppressing the HIPK3/p53 and HIPK3/Fas signaling cascades (60). Similarly, exosome-derived long non-coding RNA LINC00161 targets and inhibits miR-590-3p, thereby relieving the inhibitory effect of miR-590-3p on its downstream target gene ROCK2 and activating the ROCK2 signaling pathway. Stimulation of this pathway significantly enhances HCC angiogenesis (61). Concurrently, circRNA - 100,338 is abundantly present in metastatic hepatocellular carcinoma (HCC) cells and their exosomes. Its upregulation or downregulation notably affects HCC cell invasiveness, endothelial cell proliferation, angiogenesis, and permeability, all contributing significantly to tumor metastasis (62). In conclusion, acquiring a thorough understanding of the mechanisms driving pre-metastatic niche (PMN) formation is crucial for developing effective therapies. This knowledge will enable us to target early metastatic stages and deepen our understanding of organ-specific metastasis (63).

### 4.2 Mechanisms underlying exosome organotropic metastasis

**Figure 2:**
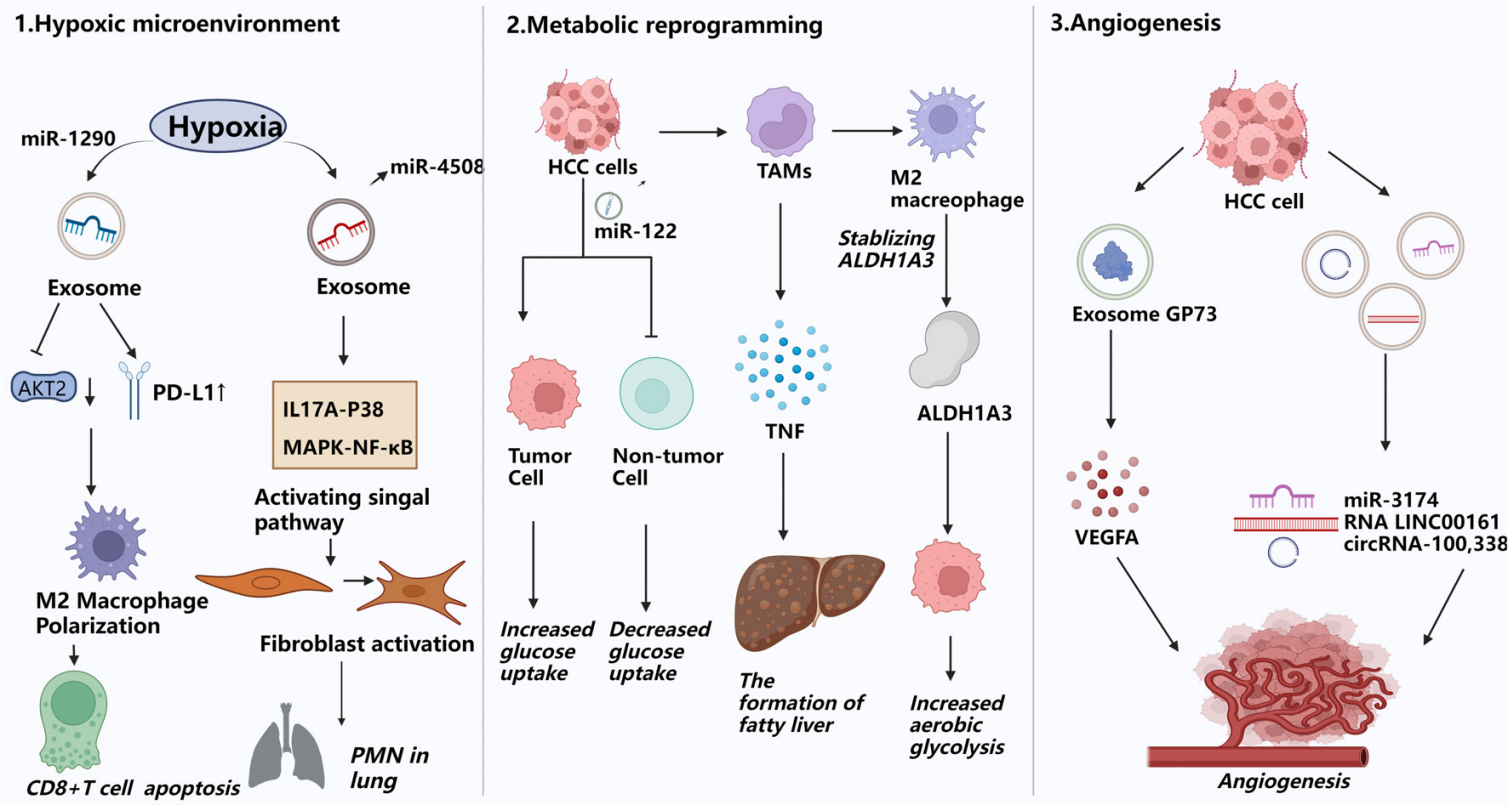
Exosomes promote pre-metastatic miche formation. This figure illustrates how exosomes facilitate the formation of the pre-metastatic niche(PMN) by remodeling the microenvironment of distant organs to create permissive conditions for tumor cell colonization and proliferation. 1) Hypoxic microenvironment modulation:Hypoxia in HCC induces exosome secretion carrying miR-1290, promoting M2 macrophage polarization, upregulating PD-L1, inducing CD8^+^ T cell apoptosis, and driving EMT, enhancing HCC cell migration and invasion. 2) Metabolic reprogramming:Cancer cells can limit glucose uptake by non-tumor cells in the pre-metastatic niche through secreting miR-122-rich vesicles. They can also use this mechanism to inhibit pyruvate kinase (PKM) in pancreatic islet β-cells, reduce insulin secretion, and disrupt glucose homeostasis, which in turn promotes tumor growth.HCC-derived palmitic acid-rich exosomes stimulate hepatic macrophages to secrete TNF, creating a pro-inflammatory milieu that suppresses fatty acid metabolism and oxidative phosphorylation in recipient cells, fostering steatosis and pre-metastatic niche formation. 3) Angiogenesis promotion:Exosomes carrying the protein GP73, as well as miR-3174, RNA LINC00161, and circRNA-100,338, drive neovascularization in distant organs, providing essential nutrient and oxygen supply routes for the survival and proliferation of metastatic tumor cells.

Since Stephen Paget proposed his hypothesis in 1889, the organotropism of pre-metastatic niche (PMN)-mediated metastasis has remained one of the most puzzling mysteries in cancer biology (64). The underlying mechanisms of this enigma may involve several factors. Firstly, Tumor exosomes can recruit immunosuppressive cells. After upregulating pro-inflammatory factors, exosomes induce a local inflammatory microenvironment that prompts tumor cells to produce chemokines and cytokines (65). These factors, together with tumor-derived exosomes, recruit TAMs, TANs, Tregs, and MDSCs to distant sites, potentially suppressing anti-tumor immunity,which may facilitate tumor cell metastasis and colonization (66). Furthermore, factors secreted by primary tumor cells can activate fibroblast-like stromal cells at pre-metastatic sites, promoting increased synthesis of the extracellular matrix (ECM) component fibronectin (67). Interestingly, recent studies have unveiled the critical role of exosomal integrins in mediating the targeted metastasis of exosomes, providing a novel perspective for deciphering the precise targeting mechanisms of tumor metastasis. The studies confirms that exosomes secreted by lung, liver, brain-tropic tumor cells preferentially fuse with resident cells in target organs (such as lung fibroblasts and liver Kupffer cells), remodeling tumor metastatic pathways by establishing pre-metastatic niches. For instance, lung-tropic exosomes can redirect the metastatic trajectory of bone-tropic tumor cells. Proteomic analysis of exosomes reveals that integrins α6β4/α6β1 are specifically associated with lung metastasis, while integrin αvβ5 is linked to liver metastasis. Targeting these integrins can significantly inhibit exosome uptake and organ-specific metastasis. Mechanistic studies show that integrin-mediated exosome uptake activates Src phosphorylation and induces the expression of pro-inflammatory S100 genes. Clinical data further indicate that the expression profile of exosomal integrins can serve as a potential biomarker for predicting organ tropism of tumors (64, 68, 69) (**Figure 2**).

## 5 Exosomes contribute to the construction of immunosuppressive microenvironment in HCC

The tumor microenvironment (TME) comprises various immune cell types, including T cells, tumor-associated macrophages (TAMs), myeloid-derived suppressor cells (MDSCs), and cancer-associated fibroblasts (CAFs).Through intercellular interactions, these cells collaborate to drive tumor progression, invasion, and metastasis (70). A growing body of research demonstrates that exosome play a pivotal role in establishing an immunosuppressive tumor microenvironment. Exosomes actively released by tumor cells into the surrounding milieu not only influence immune checkpoint function by carrying molecules like PD-L1 or regulating their trafficking, but also facilitate intercellular communication among cancer cells and reshape interactions between diverse immune cell populations within the TME (71).

### 5.1 Immune checkpoint remodeling via endosomal-exosomal networks

Immune checkpoint molecules, acting as “regulatory hubs” for immune homeostasis, possess physiological inhibitory functions that originally evolved to prevent autoimmunity. However, they are systematically hijacked by tumor cells, becoming key pathways for immune evasion. These molecules maintain self-tolerance by suppressing immune cell activity, while tumor cells act. ivate this inhibitory network through upregulating ligands like PD-L1, driving T cells into anergic and exhausted states to ultimately evade immune surveillance (72, 73). Within the identified inhibitory immune checkpoints, programmed cell death protein 1 (PD-1) and cytotoxic T-lymphocyte-associated antigen 4 (CTLA-4) are prominent regulators (74). Preclinical studies have also identified additional immune checkpoints on circulating small extracellular vesicles (sEVs), including PD-1, CTLA-4, and CD80 (75). The abnormal regulation of PD-L1 in tumor cells remains largely unclear. In the study, researchers systematically explored the endosomal transport of cell-surface PD-L1 in tumor cells. Their findings reveal that plasma membrane PD-L1 undergoes constant internalization, followed by trafficking from early endosomes to multivesicular bodies/late endosomes, recycling endosomes, lysosomes, and/or extracellular vesicles (EVs) (76). And disrupting phosphatidylinositol-4-phosphate (PI(4)P) generation or exosome complex function blocks exosomal secretion of the key immune checkpoint protein programmed death-ligand 1 (PD-L1) in tumor cells, leading to its accumulation in lysosomes (77). By comparing the latest studies, it is revealed that, the regulation of PD-L1 by immune cell-derived small extracellular vesicles (I-sEVs) and triazine compound 6J1 exhibits both opposing and complementary biological effects. PD-1/CD80 carried by I-sEVs promotes the secretion of PD-L1+ exosomes by tumor cells and reduces cell-surface PD-L1 expression, simultaneously inhibiting antigen-presenting molecules and intercellular adhesion molecules to ultimately induce an immunosuppressive “cold tumor” phenotype (78). In contrast, 6J1 blocks PD-L1 endocytic recycling by activating Rab5, leading to its accumulation in endocytic vesicles, while activating Rab27 to promote PD-L1 exosomal secretion (76). These dual mechanisms synergistically reduce membrane PD-L1 density, not only enhancing tumor cells’ sensitivity to T-cell killing but also remodeling the immune microenvironment by increasing tumor-infiltrating cytotoxic T cells and chemokine secretion.

### 5.2 Lymphocyte depletion due to exosomes

CD8 + T cells are crucial for tumor immunity and serve as primary antitumor defense cells. Tumor tissues can deplete CD8^+^ T lymphocytes through several mechanisms, decreasing their antitumor efficacy and allowing immunological escape (79). For instance, HCC cells release circCCAR1 in an hnRNPA2B1-dependent manner. The secreted circCCAR1 is then internalized by CD8+ T cells, inducing their dysfunction and consequently contributing to resistance to anti-PD-1 immunotherapy (46). Meanwhile, through encouraging the polarization of M2-type macrophages, miR-1290-containing exosomes released by HCC cells in a hypoxic environment triggers CD8^+^ T cell death (80). Exosomes also significantly influence CD4+ T lymphocyte differentiation. For instance, some exosomes promote the differentiation of naive CD4+ T cells into helper T cell (Th) subsets (e.g., Th1, Th2, Th17), while others induce the generation of regulatory T cells (Tregs). This differentiation mechanism is critical for regulating immune responses and maintaining immune homeostasis (81).

Furthermore, miR-500a-3p carried by HCC exosomes is involved in immune cell differentiation, HCC growth and invasion, and hepatic stellate cell (HSC) activation. Compared with the miR-500a-3p agonist-resistant group, the group treated with the miR-500a-3p agonist showed a significantly higher proportion of Tregs, highlighting the crucial role of miR-500a-3p in the suppressive microenvironment and T cell activity (82). Notably, aside from IL-2, extracellular vesicles (EVs) act as key intermediaries between CD4+ and CD8+ T cells. CD4+ T cell-derived EVs carry miR-25-3p, miR-155-5p, miR-215-5p, and miR-375, all of which activate CD8+ T cells. In a mouse melanoma model, these EVs have been shown to inhibit tumor progression by activating CD8+ T cells (70). Additionally, adipose-derived stem cells (ASCs) release exosomes that promote breast cancer progression. Zhu et al. investigated the effects of ASC-derived exosomes on gene and protein expression using western blotting, qRT-PCR, and RNA-seq. CD4+ T cells can internalize ASC-derived exosomes, which then promote their differentiation into Tregs (83).

Natural killer (NK) cells are integral to tumor immunosurveillance, as they directly eliminate mutant cells via death receptors and cytotoxic granules (84). However, in the tumor microenvironment (TME), NK cells often exhibit functional exhaustion, impairing their immunosurveillance capacity and enabling tumor cells to escape immune monitoring, thereby promoting tumor growth (85, 86). In HCC patients, HCC cells secrete circUHRF1 via exosomes, which inhibits natural killer (NK) cell function (87). As indicated in studies, NEAT1 in exosomes secreted by multiple myeloma cells suppresses NK cell activity and facilitates immune evasion by silencing PBX1 and recruiting EZH2 (88). In plasma exosomes from gastric cancer patients, elevated miR-552-5p expression is inversely correlated with the phenotypic distribution and receptor activation of NK cells. This miRNA reduces NK cell secretion of perforin, granzyme, and IFN-γ, and downregulates the expression of activation receptors (NKp30, NKp46, NKG2D), thereby impairing NK cell cytotoxicity.

### 5.3 Exosome-induced activation and polarization of myeloid-derived suppressor cells

Myeloid-derived suppressor cells (MDSCs) are heterogeneous myeloid cells originating from the bone marrow. Phenotypic differences in MDSCs from various cancer tissues and organs suggest that their differentiation and accumulation may be dependent on cancer cells (89). For instance, recent studies have revealed that exosomal PD-L1 promotes MDSC proliferation by activating the IL-6/STAT3 signaling pathway *in vitro*. In a CDX model, exosomal PD-L1 was shown to facilitate tumor progression and enhance MDSC expansion, highlighting its role in immunosuppressive microenvironment formation (90).

In cancer patients, particularly those in advanced stages, neutrophil accumulation in the peripheral blood is significantly increased. A high circulating neutrophil-to-lymphocyte ratio (NLR) is associated with poor prognosis in various cancers. Over the past decade, neutrophils’ role in cancer has gained considerable attention (91). In the TME, tumor-infiltrating neutrophils are capable of polarizing into anti-tumor N1 subsets that facilitate T cell-mediated tumor eradication or pro-tumor N2 subsets that serve as immunosuppressive cells (92). It has been confirmed that cancer cells induce neutrophil polarization via exosomal pathways, driving their conversion to pro-tumor phenotypes phenotypes.A research showed that exosomes derived from colorectal cancer (CRC) facilitate the circulation of PACRGL and promote neutrophil differentiation to the N2 phenotype via the miR-142-3p/miR-506-3p-TGF-β1 axis. Knockdown of CircPACRGL impairs this differentiation, which can be reversed by exosome complementation (93).

At the same time, macrophages play dual roles in cancer progression and metastasis. Pro-inflammatory M1 macrophages can phagocytose tumor cells and exert antitumor effects, whereas anti-inflammatory M2 macrophages—particularly tumor-associated macrophages (TAMs)—promote tumor growth and invasion by secreting cytokines and growth factors (94). Exosomes contain non-coding RNAs (ncRNAs), which are essential for a variety of physiological functions. Tumor-derived exosomal ncRNAs polarize macrophages toward the M2 phenotype by activating signaling pathways, regulating signal transduction, modulating transcriptional and post-transcriptional processes, and promoting the formation and progression of the tumor microenvironment (95). Upon uptake by human umbilical vein endothelial cells and HCC cells, M2 macrophage-derived exosomes stimulate angiogenesis, vascular permeability, and epithelial-mesenchymal transition (EMT). A feedback loop is established when HCC cells cocultured with M2 exosomes release increased levels of GM-CSF, VEGF, G-CSF, MCP-1, and IL-4, which recruit M2 macrophages (96). Jia et al. discovered that HCC-derived exosome circTMEM181 increases immunosuppression and resistance to anti-PD-1 reatment by upregulating CD39 expression. Targeting CD39 on macrophages can inhibit the ATP-adenosine pathway and reverse this resistance (97). Tumor-associated macrophages (TAMs) enhance the aggressiveness and biological behavior of HCC cells through multiple mechanisms. TAMs secrete exosomes containing various molecules that promote intercellular communication in HCC and modulate target cell functions. Non-coding RNAs also regulate TAM polarization in the tumor microenvironment (TME). These findings suggest that TAMs and their exosomes play a critical role in establishing the immunosuppressive microenvironment of HCC and could serve as potential therapeutic targets (98).

### 5.4 Stromal cells in the tumor microenvironment

Tumor cells have been identified for their ability to release exosomes, which have a substantial impact on the tumor microenvironment (TME). However, it is also important to recognize that stromal cells within the TME are prolific exosome producers. These stromal cells, which include cancer-associated fibroblasts (CAFs), and endothelial cells, Mesenchymal Stem Cells (MSCs) produce exosomes containing a variety of bioactive chemicals that influence both tumor cell behavior and immune cell function, thereby contributing to the formation of an immunosuppressive microenvironment.

#### 5.4.1 Cancer-associated fibroblasts

In the tumor microenvironment, cancer-associated fibroblasts (CAFs) are critical drivers of tumor progression, influencing extracellular matrix synthesis and remodeling through direct cell contact and the secretion of regulatory factors (99). According to a study, CAFs increase HCC through exosomal miRNAs. RNA sequencing and qRT-PCR analyses revealed that CAF-derived exosomes (CAF-Exo) have higher miR-92a-3p levels than normal fibroblast-derived exosomes (NF-Exo), and this miRNA promotes HCC cell migration, proliferation, and stemness. By targeting AXIN1, exosomal miR-92a-3p activates β-catenin/CD44 signaling to promote tumor growth and stemness *in vivo*. Thus, CAF-derived miR-92a-3p promotes HCC via the Wnt/β-catenin pathway, representing a potential therapeutic target (100). A similar mechanism has been observed in colorectal cancer: CAF-derived exosomes upregulate miR-92a-3p in tumor cells, activating the Wnt/β-catenin pathway and suppressing mitochondrial apoptosis via inhibition of FBXW7 and MOAP1—processes that further enhance EMT and stemness (101). Also, The function of exosomal circHIF1A from hypoxia-induced CAFs in HCC carcinogenesis was investigated in another work. exosomal circHIF1A from hypoxia-induced CAFs increased PD-L1 in a HuR-dependent manner, promoting immunological escape and HCC development (102).

Conversely, tumor-derived exosomes can induce the transformation of CAFs, thereby creating a vicious cycle that perpetuates and accelerates tumor progression. For example, HCC cells transform normal HSCs to CAFs via exosomal miRNA-21, which targets PTEN and activates PDK1/AKT signaling in HSCs. Activated CAFs release angiogenic cytokines (VEGF, MMP2, MMP9, bFGF, and TGF-β) that promote cancer growth. Clinically, increased serum exosomal miRNA-21 levels are associated with CAF activation and vascular density in HCC patients (103). Exosomes derived from hepatocellular carcinoma stem cells, which contain circ-ZEB1 and circ-AFAP1, also facilitate crosstalk between cancer stem cells (CSCs) and non-CSCs. These circRNAs enhance stemness and epithelial-mesenchymal transition (EMT) in HCC cells, resulting to more aggressive tumor behavior and a worse prognosis (104). Moreover, exosomes carrying HSPC111 can activate HSCs into CAFs, thereby supporting pre-metastatic niche formation and facilitating liver metastasis in CRC (105).

#### 5.4.2 The edothelial cells and platelets

Hepatocellular carcinoma (HCC) is an extremely vascular cancer. Exosomes produced during tumor progression can influence vascular endothelial cell proliferation, migration, and tube formation, reshaping the tumor vascular network (106). Previous studies found that endothelial progenitor derived exosomes promote vascular repair in a rat balloon injury model by enhancing endothelial cell proliferation, migration, and tube formation via miR-21-5p-mediated suppression of THBS1 (107). Also, endothelial progenitor derived exosomes accelerated re-endothelialization in arteries and enhanced endothelial cell proliferation and migration *in vitro*, highlighting their potential for vascular repair (108). These studies reveal the dual role of exosomes in vascular repair and tumor angiogenesis, laying the foundation for further exploring their functions within the tumor microenvironment.

Likewise, exosomes from HCC cells can influence endothelial cells, effectively reprogramming them to promote angiogenesis. For example, miR-210 is highly expressed in exosomes from HCC patients and hepatoma cells. It enhances endothelial cell tubulogenesis *in vitro* and angiogenesis *in vivo*, correlating with increased microvessel density in HCC tissues. Mechanistically, exosomal miR-210 targets SMAD4 and STAT6 in endothelial cells (109). In the same time, HCC cells secrete small extracellular vesicles carrying von Willebrand factor (sEV-vWF), which stimulate endothelial cells to secrete fibroblast growth factor 2 (FGF2). FGF2 then binds to fibroblast growth factor receptor 4 (FGFR4) on HCC cells, activating the ERK1 pathway to promote HCC cell proliferation and forming a positive signaling loop between tumor cells and endothelial cells (106). Interestingly, it has been revealed that endothelial cell-derived EVs in the tumor environment can recruit macrophages and induce an immunomodulatory state that promotes tumour growth.Using RNA-Seq, the researchers discovered miRNAs (miR-142-5p, miR-183-5p, and miR-222-3p) in endothelium EVs that regulate immunological pathways. In animal models, miRNAs delivered to macrophages via EVs polarize them to an M2-like phenotype, which promotes tumor growth (110).

Circulating platelet microparticles (PMVs) with procoagulant properties contribute to thrombotic diseases such as cancer by enhancing prothrombinase assembly sites and recruiting active tissue factor (TF). They may serve as accessible hypercoagulability biomarkers and potential thrombosis management targets (111). Additionally, cancer cells, similar to inflammatory cells, can induce thrombocytosis by secreting pro-inflammatory cytokines that stimulate megakaryocytopoiesis and platelet production. Secreted PF4 promotes bone marrow megakaryocyte proliferation, elevating platelet counts to accelerate tumor growth. Tumor-derived exosomes activate platelets to initiate thrombin generation, shifting platelets from tissue repair “orchestrators” to cancer progression “accomplices” by carrying metastatic cells and facilitating metastasis (112). In the tumor microenvironment, platelets and endothelial cells reciprocally interact. Platelet-derived exosomes can upregulate VEGF mRNA expression in lung cancer cells and promote their adhesion to endothelial cells (113). These findings illustrate the intricate interactions that occur between endothelial cells and platelets in the tumor microenvironment, emphasizing the vital roles that these cells play in promoting dissemination and tumor growth.

#### 5.4.3 Mesenchymal stem/stromal cells may exert atitumor efects

Mesenchymal stem/stromal cells (MSCs), first identified in the bone marrow in 1976, have since been detected in nearly all human tissues. They can differentiate into various cell types, including osteoblasts, chondrocytes, and adipocytes. In addition to their ability to home to tumor sites, MSCs exhibit immunomodulatory properties, helping to suppress excessive immune responses and maintain immune homeostasis (114). MSCs-derived EVs play a pivotal role in intercellular communication and have been shown to exert antitumor effects (115). For instance, MSC-derived exosomes carrying miR-15a have been found to induce apoptosis and inhibit the proliferation, migration, and invasion of hepatocellular carcinoma (HCC) cells (116). Similarly, exosomes secreted by adipose-derived MSCs can be engineered to deliver miR-122 to HCC cells. This delivery modulates the expression of miR-122 target genes in cancer cells, thereby enhancing their sensitivity to chemotherapeutic agents and improving the *in vivo* efficacy of sorafenib (117). In summary, while exosomes secreted by most cells within the tumor microenvironment tend to support tumor progression, those derived from MSCs demonstrate antitumor activity. This effect is likely attributable to the unique immunomodulatory functions and pluripotent nature of MSCs. Since exosomes reflect the properties of their parent cells, MSC-derived exosomes are enriched with antitumor molecules and can inhibit tumor progression, distinguishing them from exosomes of other cell types, which generally promote tumor growth.

**Figure 3:**
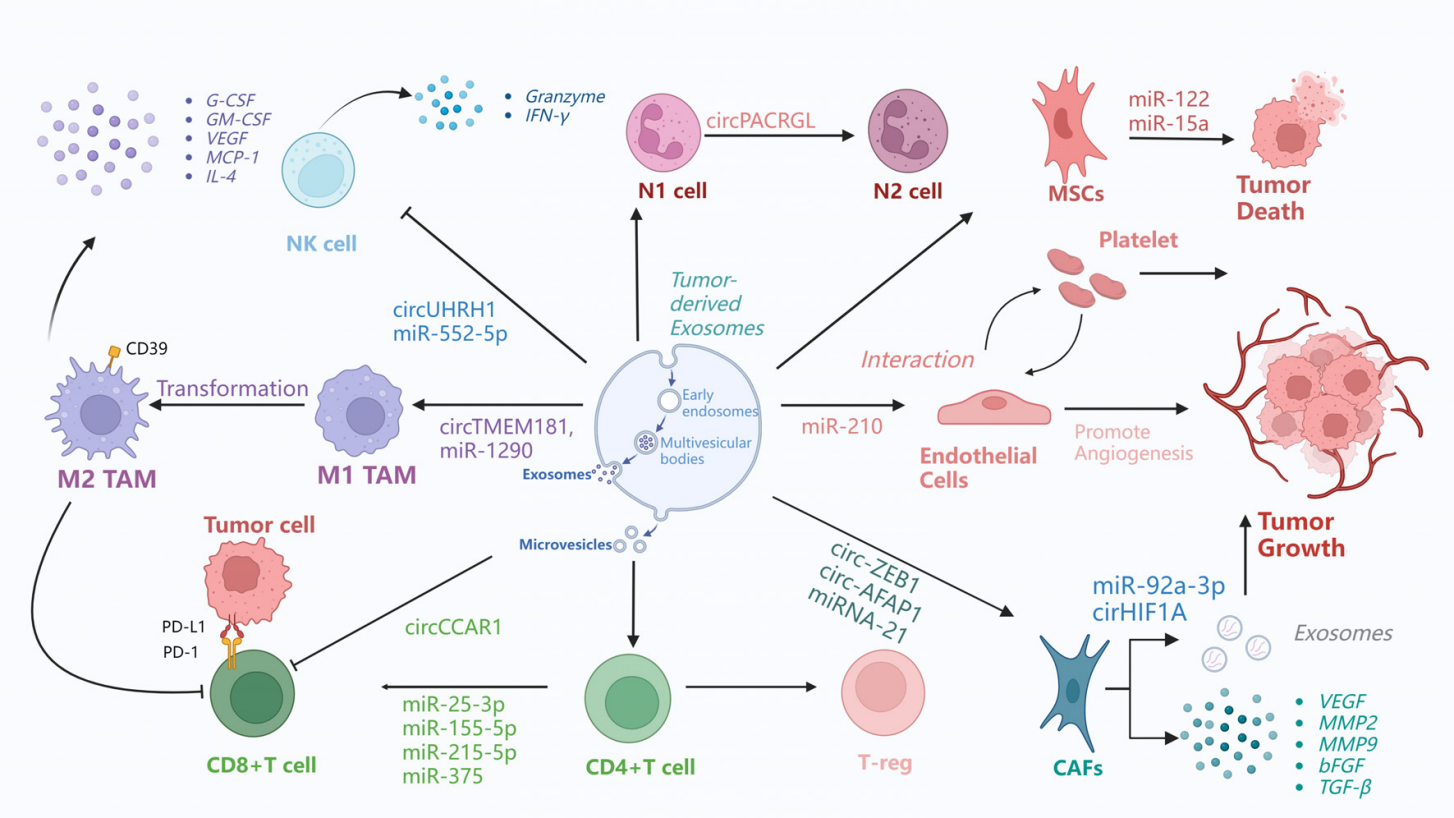
Exosome promotes tumor immunosuppressive microenvironment. This figure demonstrates the exosome-mediated interactions between different cells in the tumor microenvironment and the related immunomodulatory processes. 1) Tumor-associated macrophages (TAMs) exist in both M1 and M2 phenotypes.M1 to M2 conversion.M1 TAMs can be converted to M2 TAMs through exosome-carried circTMEM181 and miR-1290. Then, M2 TAMs secrete cytokines such as G-CSF, GM-CSF, VEGF, MCP-1, IL-4, etc. 2) Natural killer (NK) cells release circUHRH1, miR-552-5p through exosomes that act on other cells and affect the tumor microenvironment.NK cells also release granzyme and interferon-γ. 3) N1 cells can be transformed into N2 cells by exosome-borne circPACRGL. 4) MSCs receive exosomes from other cells, such as exosomes carrying miR-122, miR-15a, which can induce tumor death. 5) Exosomes from endothelial progenitor cells exert their effects by inhibiting THBS1 via miR-210, and miR-210, which is highly expressed in HCC cell-derived exosomes, targets SMAD4 and STAT6 in endothelial cells to promote angiogenesis. In addition, tumor-derived exosomes activate platelets to promote coagulation and metastasis, while platelet exosomes can enhance VEGF mRNA expression in cancer cells and their adhesion to endothelial cells. These findings demonstrate the intricate interactions between endothelial cells and platelets in the tumor microenvironment and their important roles in promoting tumor dissemination and growth. 6) CAFs are key drivers of tumor progression. They exert their effects through exosomes: for instance, miR-92a-3p in CAF-derived exosomes can target AXIN1 to activate the β-catenin/CD44 signaling pathway in HCC, and in colorectal cancer, it can activate the Wnt/β-catenin pathway while inhibiting mitochondrial apoptosis by suppressing FBXW7 and MOAP1, thereby enhancing epithelial-mesenchymal transition (EMT) and stemness. Additionally, circHIF1A in exosomes derived from hypoxia-induced CAFs can upregulate PD-L1 in a HuR-dependent manner, promoting immune escape and the development of HCC.CAFs receive exosome-carried miRNA-21, circ-ZEB1, circ-AFAP1.CAFs secrete VEGF, MMP2, MMP9, FGF, and TGF-β, which affect th TME.7)Tumor cells associated with T cells.Tumor cells interact with CD8^+^ T cells through microvesicles.PD-L1 on the surface of tumor cells binds to PD-1 on the surface of CD8^+^ T cells.CD8^+^ T cells receive exosomes carrying miR-25-3p, miR-155-5p, miR-215-5p, miR375. CD4^+^ T cells can be transformed into regulatory T cells.

The immunomodulatory and regenerative qualities of mesenchymal stem cells (MSC) make them popular in cell treatment, and their paracrine activity is essential to their effectiveness. In experimental models, it has been demonstrated that the substances that MSCs produce through extracellular vesicles—particularly exosomes—dominate their therapeutic benefits. Compared to MSC itself, MSC-derived exosomes (MSC-Exos) offer greater therapeutic benefits and far less side effects, such as toxicity from infusion (118). For example, a Phase I clinical trial is testing exoASO-STAT6 (CDK-004) for the treatment of advanced HCC and colorectal cancer liver metastases. ExoASO-STAT6 is an exosome-based therapeutic drug that converts tumor-associated macrophages (TAMs) from an immunosuppressive M2 phenotype to a proinflammatory M1 phenotype by delivering antisense oligonucleotides (ASOs) targeting STAT6 to enhance antitumor immune responses.A recent study found that this medication dramatically slowed tumor growth and even achieved complete remission, while also demonstrating notable inhibitory effects on liver metastases. (NCT05375604). In another study, seven COVID-19 patients underwent a preliminary trial of MSC exosome nebulization therapy. This therapy also achieved complete remission in a mouse model, offering new therapeutic hope for patients with severe pneumonia. The results showed that the therapy was safe and effective, with no acute allergic reactions, promoted the absorption of lung lesions, and shortened the hospitalization time of patients with mild symptoms (ChiCTR2000030261) (119). Exosomes hold great potential for treating diseases, but their clinical use in the management of HCC is still in its early stages, and even fewer clinical trials are being conducted at this time. Key challenges in the application of exosomes for HCC and other cancer treatments include: 1) Effectively enhancing cell-specific delivery by loading exogenous drugs, siRNAs, shRNAs, or miRNAs into exosomes; 2) Extending the *in vivo* half-life of bioengineered exosomes or exosomal vaccines to prevent rapid elimination by the kidneys, liver, or immune cells; 3) Overcoming technical difficulties in producing clinical-grade exosomes and ensuring quality control of exosomes administered to patients (**Figure 3**).

## 6 Exosome-mediated clinical detection of HCC and prospects for engineered drug delivery applications

### 6.1 Exosomes in clinical detection of cancer

The majority of eukaryotic cells produce exosomes, a form of extracellular vesicle that is essential for intercellular communication. These vesicles carry numerous bioactive molecules, including proteins, DNA, mRNA, microRNAs (miRNAs), long non-coding RNAs (lncRNAs), and circular RNAs (circRNAs). Exosomal cargo plays a major role in tumor growth, metastasis, and angiogenesis over the course of cancer, and these elements have potential as predictive biomarkers in cancer patients (120). **Table 1** summarizes clinical trials focused on exosome-based detection of digestive system cancers.In particular, exosomal miRNAs have demonstrated diagnostic potential for HCC (121). For example, Wang et al. identified a panel of exosomal miRNAs—miR-122, miR-21, and miR-96—that could serve as reliable diagnostic biomarkers for HCC (122). Furthermore, reduced expression of exosomal miR-23a-3p has been found to be negatively correlated with overall survival in HCC patients, suggesting its potential for evaluating prognosis and treatment response (123). Although the clinical application of such RNA-based biomarkers for HCC screening remains to be fully validated, their potential is considerable. Exosomes have already been employed in clinical trials to detect malignancies within the digestive system, underscoring their diagnostic promise. However, data specific to exosome-based identification in HCC are currently limited. Continued research in this area is anticipated to yield significant breakthroughs, advancing early detection and precision therapy for HCC.

**Table 1:** Clinical trials related to the identification of digestive system cancers using exosomes.

NCT number	Study title	Study status	Summary	Study type
NCT06388967	Pancreatic Cancer Detection Consortium	RECRUITING	Prospectively validate an exosome - based miRNA signature for non - invasive early pancreatic ductal adenocarcinoma detection.	OBSERVATIONAL
NCT06342427	Stomach Cancer Exosome - based Detection	COMPLETED	Develop and validate a blood assay for non - invasive gastric cancer detection.	OBSERVATIONAL
NCT06342401	Early Onset Colorectal Cancer Detection	RECRUITING	Use a blood - based microRNA assay for non - invasive early - onset colorectal cancer detection.	OBSERVATIONAL
NCT05427227	Prospectively Predict the Efficacy and Explore the Mechanism of Treatment of Gastrointestinal Tumors Based on Peripheral Multi - omics Liquid Biopsy	Unknown status	Do dynamic multi - omics detection of plasma - derived exosomes to study treatment efficacy and mechanism for gastrointestinal cancer.	OBSERVATIONAL
NCT06023121	Use of a Liquid Biopsy Signature to Detect Early - onset Gastric Cancer	COMPLETED	Identify novel bioindicators for early - onset gastric cancer detection.	OBSERVATIONAL
NCT06342440	Early Detection of Advanced Adenomas and Colorectal Cancer	RECRUITING	Develop a blood assay for early detection of colorectal adenomas and cancer using machine learning and biological analyses.	OBSERVATIONAL
NCT06654622	Exosome - based Detection of Molecular Residual Disease in Stage II - III Colorectal Cancer	RECRUITING	Establish an exosome - based liquid biopsy signature to detect molecular residual disease in stage II - III colorectal cancer patients for personalized treatment.	OBSERVATIONAL
NCT05625529	ExoLuminate Study for Early Detection of Pancreatic Cancer	RECRUITING	Compare ExoVerita assay with standard - of - care methods for early pancreatic cancer detection in high - risk patients.	OBSERVATIONAL

Notably, multiple studies have constructed and validated hepatocellular carcinoma prognostic models based on exosomal mRNA using multiple databases, identifying key genes that not only have diagnostic value but are also closely associated with immunotherapy and the tumor microenvironment (124). For example, one study addressed the challenge of prognostic evaluation in HCC by exploring the value of a risk prognostic model based on blood exosomal mRNA. From the TCGA and exoRBase 2.0 databases, 44 prognosis-related genes were screened, and 6 exosomal risk genes including CLEC3B were selected through analysis to construct the model. Validation showed that the model’s risk score was a robust independent prognostic factor, and the nomogram model incorporating pathological stage yielded the best clinical benefits. This study is the first to confirm that these 6 genes exist in blood exosomes of HCC patients, enabling liquid biopsy to avoid puncture. These genes originate from multiple cell types, potentially serving as diagnostic markers, and the high-risk group may benefit from immunotherapy (125) Additionally, A 4-gene prognostic signature (DYNC1H1, PRKDC, CCDC88A, and ADAMTS5) related to exosomal mRNA was constructed and validated. A prognostic nomogram based on this signature exhibits prognostic ability for HCC. The genes in the signature are involved in the extracellular matrix, ECM-receptor interaction, and PI3K-Akt signaling pathway. Their expression is positively correlated with immune cell infiltration in the TME (126).

### 6.2 Exosome-based drug carrier construction

Researchers are interested in creating engineered exosome nanocarriers because exosomes are important for cellular communication and, as naturally occurring nanocarriers, they are not only low-immunogenic and biocompatible but also have a lot of potential for pharmacotherapy and drug targeting (127). **Table 2** presents a comparison of Natural Exosomes, Engineered Exosomes, and Traditional Carriers.

**Table 2:** Comparison of natural exosomes, engineered exosomes, and traditional carriers.

Feature	Natural exosomes	Engineered exosomes	Traditional carriers (e.g., liposomes, polymers)
Biocompatibility	High – derived from cells, inherently biocompatible (173)	High – retain natural exosome properties even after engineering (174)	Low to Moderate – depends on material, some may cause toxicity (175, 176)
Immunogenicity	Low (177)	Low – surface modifications do not significantly increase immunogenicity (174)	Variable – some synthetic carriers may trigger immune responses (178, 179)
Targeting Ability	Limited natural targeting capability (180)	Enhanced – can be modified with antibodies (e.g., EGFR) or ligands for specific tumor/tissue targeting (130, 180)	Modifiable – targeting possible but often less specific or efficient
Cargo Protection	Good – protect nucleic acids and proteins naturally (181)	Excellent – preserve cargo integrity with added targeting benefits (182, 183)	Variable – some methods may damage cargo or reduce stability (184)
Clinical Potential	Emerging – limited by targeting and scalability	High – demonstrated success in tumor targeting, liver disease therapy, and increased drug sensitivity (185)	Established – some FDA-approved drugs, but limited by side effects

**Figure 4:**
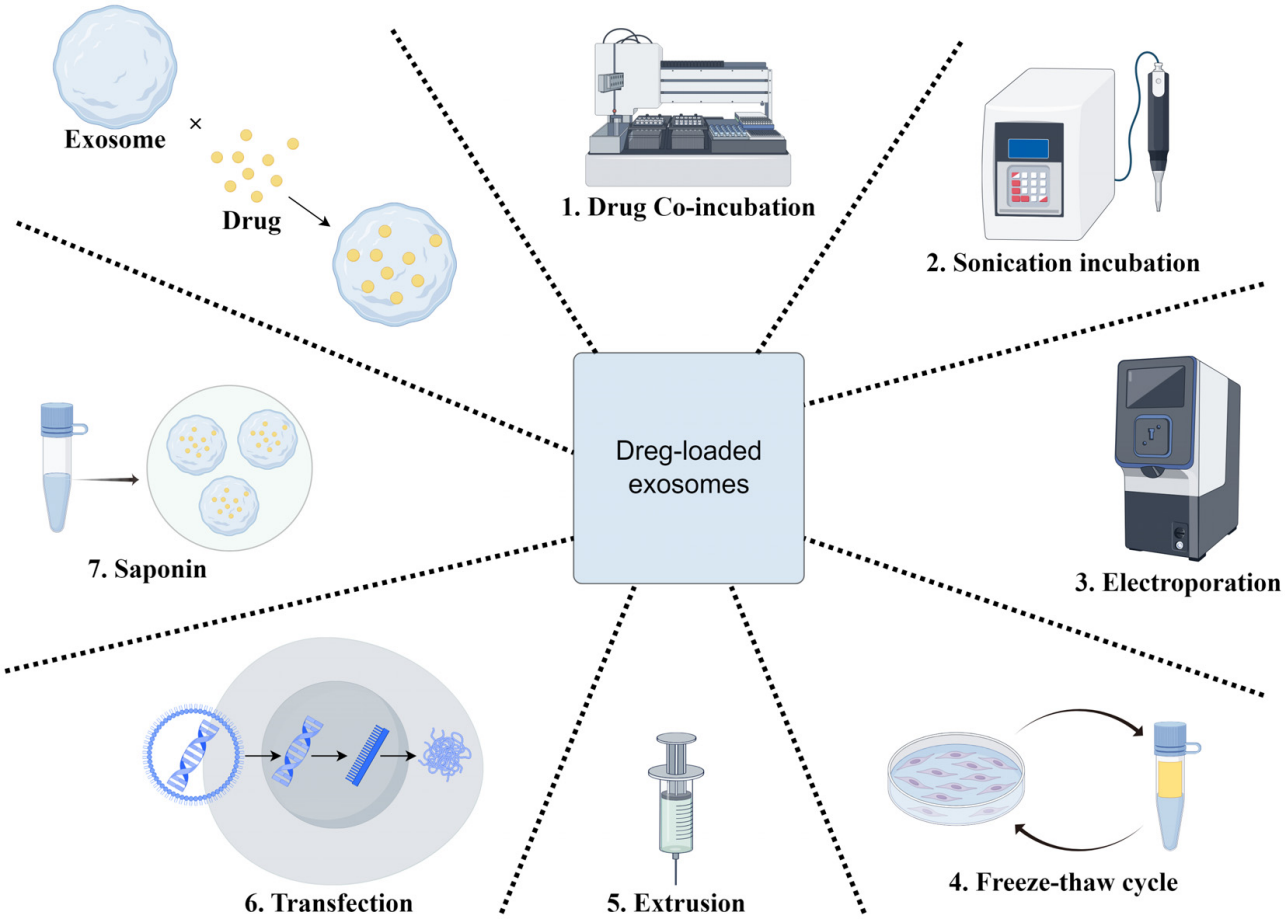
Engineered exosome drug delivery system construction. This figure shows the preparation of drug-loaded exosomes through seven key steps to boost drug loading efficiency and exosomal stability for effective therapy. 1) Drug co-incubation:Exosomes are co-incubated with therapeutic agents to enable drug adsorption or internalization, facilitating initial drug binding to the exosomal membrane. 2) Ultrasonication:Ultrasonication treats the exosome-drug mixture, enhancing drug penetration into the exosomal membrane and improving loading capacity. 3) Electroporation:Electric fields create transient pores in the membrane, driving drug molecules into the exosomal lumen, especially suitable for hydrophilic drugs. 4) Freeze-thawing cycle:Alternating freezing and thawing increases membrane fluidity and permeability, promoting drug incorporation. 5) Extrusion:Exosomes are forced through narrow channels or membranes, enhancing membrane deformability and encapsulation efficiency via mechanical stress. 6) Transfection:Liposomes or other reagents deliver therapeutic RNA into exosomes, suitable for loading genetic materials. 7) Saponin-mediated loading:Saponin disrupts cell membranes to facilitate drug uptake by exosomes, enhancing internalization for novel nanoscale drug delivery systems.

On the one hand, exosome targeting can be enhanced by surface modification (128, 129), thus enabling the precise treatment of specific tumor sites or tissues. For example, by enhancing targeting, it can be used to treat brain, breast, lung, liver, colon tumors, and heart diseases (129). By modifying the exosome surface with an EGFR antibody, Ohno et al. and Kooijmans et al. found that exosomes could be targeted to EGFR-expressing tumor cells (130). Tamura et al. used electrostatic interactions to modify exosomes with cationic branched-chain amyloid polysaccharides, which increased their uptake in HepG2 cells considerably. Unlike unmodified exosomes, modified exosomes enter cells via the desialylated glycoprotein receptor, which is exclusively expressed by hepatocytes. When modified exosomes were intravenously administered into animals with concurrent sabinoglobulin A-induced liver injury, they accumulated in the liver tissues and dramatically increased the anti-inflammatory effects (131). The use of exosomes as drug carriers has attracted much attention. Owing to their non-immunogenicity, low toxicity, and biocompatibility, they are considered ideal drug delivery vehicles (132). For example, Mukhopadhya et al. demonstrated, through the application of high-performance liquid chromatography (HPLC), that the electroporation method successfully encapsulated 90.1 ± 12 μg (36% of the available adriamycin) and 68.0 ± 10 μg (27%) of the drug into MSCs and milk exosomes, respectively, in a comparative study of drug loading into milk and MSC exosomes. Using imaging flow cytometry, they confirmed that this method did not compromise the integrity of exosomal surface proteins, whereas the sonication method significantly reduced the number of CD9+/CD63+ exosomes (p<0.0001), thus validating the efficacy and safety of exosomes as drug carriers (133). BM-MSC exosomes have also made significant progress in the treatment of HCC as drug carriers. BM-MSC-derived exosomes delivering the nucleic acid drug siGRP78 enhanced the sensitivity of HCC cells to sorafenib, thereby improving the drug effect (134). Extracellular vesicle (EV) proteomics analysis can help predict therapy response. In a rare clinical trial, 25 patients with advanced HCC were treated with selective internal radiation therapy (SIRT) in combination with sorafenib, while 20 patients received sorafenib alone. The results revealed that patients with greater EV-GPX3/ACTR3 levels and lower EV-ARHGAP1B levels had superior efficacy when treated with SIRT in conjunction with sorafenib (AUC = 1) (135) (**Figure 4**).

### 6.3 Exosomal constraints in cancer therapy and corresponding strategies

#### 6.3.1 Technical challenges posed by exosome heterogeneity

Exosomes exhibit striking similarities in their physicochemical properties, as well as in the density, charge, and other characteristics of the biomolecules they carry. This makes it challenging for separation techniques based on traditional physical parameters—such as ultracentrifugation and ultrafiltration—to accurately distinguish functionally distinct exosome subpopulations (19). Within the tumor microenvironment, exosomes derive from an extremely complex range of biological sources, including epithelial cells, tumor cells, and various immune cells (e.g., dendritic cells, B cells, T cells, and mast cells). The functions of these exosomes are often antagonistic to each other (136, 137). However, contemporary technological limitations hinder the effective differentiation of exosomes originating from various cellular sources, complicating experimental efforts to accurately distinguish them. This difficulty in differentiating cellular origins directly contributes to imprecision in attributing functions to exosomes in research, making it challenging to determine which specific exosome subpopulation is responsible for particular functions—such as promoting cancer progression or inhibiting tumor growth.

#### 6.3.2 Comparison of different separation techniques and standardization issues

Different exosome isolation techniques have their own advantages and limitations. Studies have shown that ultracentrifugation is often regarded as the “gold standard (138–140). However, in practice, it is complicated and time-consuming, requires expensive ultracentrifuges, and requires a high level of operator skill (141). In contrast, ultrafiltration filters biological samples through a filter membrane with a specific pore size (usually ≤ 100 nm), trapping exosomes on the membrane. It is relatively simple to operate, suitable for large-scale sample processing, and inexpensive. However, impurities may be introduced, requiring further purification, and exosomes may aggregate on the filter membrane, affecting recovery (142). The immunocapture method uses specific antibodies to capture exosomes via surface proteins (e.g., CD63 and CD81), with separation achieved using magnetic beads or microtiter plates.This technique is appropriate for researching exosomes released by particular cell types since it is very specific and can separate exosomes from particular sources. Its drawbacks include its high cost, the requirement for well-known cell surface markers, and the possibility that antibody affinity may have an impact on capture efficiency (143, 144). The density gradient separation method utilizes a density gradient (e.g., sucrose gradient) to separate exosomes, where exosomes of different densities are layered in the gradient. The advantage of this method is the high purity of the separation and the ability to obtain high-quality exosomes, which is particularly suitable for studies that require high-purity exosomes for subsequent biochemical analysis. However, one disadvantage of this strategy is that it is difficult and time-consuming (145, 146). Commercial kits offer a more convenient solution than traditional isolation methods. Exosomes are isolated via precipitation and captured using specific chemical reagents or nanomaterials. While the advantages of kits include ease of operation, suitability for routine laboratory use, and higher purity in some cases, they also have drawbacks such as higher cost. Additionally, separation efficiency and purity may vary by brand and method; thus, selection should be evaluated based on specific needs (147, 148). In recent years, microfluidics, a high-precision separation technology based on microfluidic chips, has been used to separate exosomes using microchannels and physical fields (e.g., electric and magnetic fields). This technology enables the efficient capture and separation of exosomes by precisely controlling the fluid flow in microchannels. Microfluidic chips are typically composed of multilayered materials containing a complex network of microchannels that match the size of exosomes, thereby enabling highly selective separation. Microfluidic technology has the advantages of high throughput, high automation, and high purity, and can process multiple samples simultaneously, significantly improving the separation efficiency and making it suitable for the high-throughput screening of large-scale samples. In addition, the microfluidic system can realize automated operation, reduce human error, and improve the repeatability and reliability of the experiments. By precisely controlling the fluid flow and physical field, microfluidics can provide high-purity exosomes suitable for subsequent biochemical analyses and functional studies. However, microfluidic technology has high equipment costs, requires specialized personnel for operation and maintenance, and requires a high level of technical skills and personnel training in the laboratory. Another study proposed a new method for exosome isolation and detection by combining an automated centrifugal microfluidic disc system (exo-CMDS) with an aptamer fluorescence system (exo-AFS). The exo-CMDS efficiently isolates 5.1 × 10^9^ particles/mL of exosomes from <300 μL of blood within 8 minutes, enabling lung cancer diagnosis with 91% accuracy (147, 149–151).

Furthermore, variations in exosome isolation protocols across laboratories have resulted in significant differences in data from exosome marker studies.Such differences weaken the reliability and comparability of the study results and increase uncertainty and complexity in clinical applications. For example, different isolation methods may lead to inconsistent expression levels and functional properties of exosomal markers, making clinical decision-making more challenging. Thus, the lack of standardization hinders the progress of exosome marker research and poses a challenge for the establishment of clinical diagnosis and treatment plans based on exosomes (152). For instance, ADSC-derived exosome protein expression varies with different isolation methods and lipopolysaccharide (LPS) treatment (153). **Table 3** presents a comparison of different exosome isolation and purification methods.

**Table 3:** Comparison of different exosome isolation and purification methods.

Separation method	Separation principle	Advantages	Disadvantages	Reference
Ultracentrifugation	Uses high centrifugal force to separate exosomes based on size and density	Gold standard for exosome isolation, high purity, widely used	Complex operation, expensive, time-consuming, requires skilled personnel	(138–140)
Ultrafiltration	Filters biological samples using a specific pore size membrane to capture exosomes	Simple operation, low cost, suitable for large sample processing	Potential impurities, requires further purification, aggregation on the membrane may affect recovery	(142)
Immunocapture	Uses specific antibodies to capture exosome surface proteins	Highly specific, ideal for isolating exosomes from specific cell types	Expensive, requires known surface markers, capture efficiency depends on antibody affinity	(143, 144)
Density Gradient Separation	Uses density gradients (e.g., sucrose) to separate exosomes based on their density	High purity exosome separation, suitable for biochemical analysis	Complex operation, time-consuming	(145, 146)
Commercial Kits	Uses chemical reagents or nanomaterials for precipitation or capture of exosomes	Convenient and easy to use, provides rapid exosome isolation	High cost, efficiency and purity may vary depending on the brand and method	(147, 148)
Microfluidic Technology	Uses microfluidic chips with microchannels and physical fields (electric/magnetic fields) to separate exosomes	High throughput, automation, high purity, suitable for large scale screening	High equipment cost, requires professional staff, maintenance and operation can be complex	(147, 149–151)

#### 6.3.3 Challenges and innovative solutions for exosome production

Studies have revealed that fewer than 1μg of exosomal proteins may be extracted per 1 mL of culture medium. However, the effective dose required for clinical therapy is typically 10–100 μg, making it challenging to meet the actual clinical demand for exosome yield (154, 155). Promisingly,edible plant-derived exosome-like nanoparticles (PELNs) provide a viable solution to the challenge of exosome yield. Characterized by high yield, low cost, ethical compatibility, and numerous health benefits, PELNs are well-positioned to overcome the technical hurdles faced by mammalian nanoparticles (156). Exosome - like nanovesicles (MLNPs) derived from fresh Morus alba leaves comprise lipids, proteins, and flavonoids. Among these components, galactose can specifically target liver tumors. MLNPs exhibit stability under gastrointestinal conditions and possess biocompatibility for *in vivo* applications. *In vitro* studies revealed that MLNPs were selectively internalized by Hepa1–6 cells, where they inhibited cell proliferation and migration and induced apoptosis. In mouse HCC models, orally administered MLNPs demonstrated effective liver targeting, suppressed tumor growth, modulated the gut microbiota, and were found to be biosafe (157). Additionally, blueberry-derived edible nanoparticles (BELNs) can mitigate rotenone-induced apoptosis in HepG2 cells via their antioxidant properties. They also alleviate insulin resistance and hepatic impairment by reducing AST and ALT levels, while decreasing lipid droplet accumulation by suppressing the expression of FAS and ACC1—two key transcription factors involved in hepatic *de novo* lipogenesis in high-fat diet (HFD)-fed mice.Collectively, it suggested that BELNs hold potential as a novel antioxidant for the management of NAFLD (158). Besides, a recent study also reported that milk-derived extracellular vesicles provide a relatively low-cost and scalable platform for large-scale mEV production (159).

#### 6.3.4 Advances in standardized and implementation of exosomes

Meanwhile, advancing the establishment of standardized operational protocols is pivotal for overcoming critical bottlenecks in exosome research and translational applications. Uniform procedures for isolation,characterization, and quality control are essential to ensure reproducible results and bridge the gap between bench research and clinical translation (160, 161).

Membrane-associated proteins and lipids are central to exosome structure and function, and can be precisely analyzed using membrane proteomics, lipidomics, and biotin labeling combined with mass spectrometry (162). In HCC, the marker proteins frequently detected in exosomes include the four transmembrane protein family (CD9, CD63, CD81, and CD82). Additionally, exosomes contain membrane transporter proteins (e.g., Rab GTPases and Annexins), heat shock proteins (e.g., HSPA8 and HSP90), and other common proteins, such as Alix and TSG101 (163). For instance, Kowal and colleagues performed proteomic analysis to characterize diverse EV subpopulations. They employed antibody-conjugated beads against CD9, CD63, or CD81 for immunoisolation of distinct EV subtypes from cell culture supernatants, enabling comparative proteomic profiling of these heterogeneous populations (143).

Intraluminal constituents, including RNAs, DNAs, and proteins, require membrane disruption through detergent-based lysis (e.g., n-dodecyl β-D-maltoside, Triton X-100, Digitonin) or iterative freeze-thaw cycles for downstream analysis (164). Detergents act by solubilizing lipid bilayers: non-ionic surfactants (e.g., Triton X-100) preserve protein functionality, while ionic detergents (e.g., SDS) enhance lysis efficiency but cause protein denaturation. In contrast, freeze-thawing uses physical forces from ice crystal formation to disrupt exosomal membranes.Both strategies effectively release encapsulated cargoes for subsequent characterization via RNA-seq, mass spectrometry, or Western blotting (165). Through differential gene expression and pathway enrichment analyses of public RNA-seq datasets, 30 dysregulated genes in HCC tissues were identified, narrowed to 10 key genes via protein-protein interaction analysis. Excluding POLD1 and MCM4 due to poor performance in additional datasets, CDK1, FEN1, and PCNA were found significantly elevated in plasma exosomes of HCC patients compared to non-HCC individuals (HBV-infected hepatitis patients and healthy controls) (166). For protein content, mass spectrometry (MS) enables systematic identification of intraluminal proteins through highly sensitive detection methods (167). Research showed that exosomal proteomics-driven discovery and multi-modal validation identify RAB13 as a pivotal regulator of HCC metastasis (168).

For the digital identification of target exosomes, researchers have developed a droplet microfluidics-based immunosorbent assay. By sandwiching enzyme-linked immunosorbent assay (ELISA) complexes on magnetic microbeads and encapsulating the exosomes into microdroplets for single exosome counting, the researchers were able to count cancer-specific exosomes with a detection limit as low as 10 enzyme-labeled exosome complexes per microliter (~10-_17_ M). The experiment successfully applied this platform to plasma samples from patients with breast cancer, demonstrating its potential for early cancer diagnosis and biomarker discovery (169). In addition, single-EV analysis technologies (e.g., MASEV) enable multiplexed protein analysis of a single exosome, facilitating early cancer diagnosis and organ origin tracking (170).

Additionally, exosomes are typically identified using a variety of methods, including transmission electron microscopy (TEM) to observe their morphological features, nanoparticle tracking analysis (NTA) to measure their particle size and concentration, western blotting (WB) to detect exosome-specific protein markers, and polymerase chain reaction (PCR) to analyze their nucleic acid composition. Each of these methods has its own advantages and disadvantages. For example, TEM is complex and involves extensive sample preparation, NTA necessitates rigorous sample purity and dispersion, WB results can be influenced by antibody specificity, and PCR has excellent sensitivity but necessitates high-quality nucleic acid extraction. Therefore, in practical identification, it is vital to select appropriate methods based on research goals and sample characteristics, and to combine multiple approaches to improve the accuracy and reliability of results (13, 39, 162, 171, 172).

## 7 Conclusion

This review offers a comprehensive overview of the mechanisms by which exosomes function in HCC, including their roles in tumor treatment resistance, metastasis, and tumor microenvironment remodeling, alongside a discussion of their potential clinical applications.As key mediators of intercellular communication, exosomes transport a range of bioactive molecules that markedly regulate the biological behavior of tumor cells and play a pivotal role in the initiation, progression, and drug resistance of HCC.Furthermore, the engineered alterations and drug-carrying capacity of exosomes present new ideas for future therapeutic techniques, potentially leading to novel pathways for HCC detection and therapy. Exosomes can cooperate with immunotherapy; for example, exosomes produced from CAR-T cell therapy are expected to decrease major side effects, such as cytokine storms. CAR-T cell exosomes are thought to offer a promising way to overcome the challenges of CAR-T therapy for solid tumors because they inherit the targeting and immune activation abilities of CAR-T cells, as well as their high tissue penetration.

Despite the remarkable progress in elucidating the mechanisms of exosome action in HCC, our study is not without limitations. The majority of existing research has primarily focused on *in vitro* and cellular models, with limited validation through *in vivo* studies. Additionally, clinical investigations into the therapeutic and diagnostic applications of exosomes remain sparse. The efficacy and impact of exosome-based treatments are challenging to quantify due to the restricted scale and scope of current trials. Moreover, there is a significant lack of authoritative data supporting the detection of these extracellular vesicles. The mechanisms underlying exosome biosynthesis and secretion, as well as their functional roles within the tumor microenvironment, remain to be fully elucidated. The precise mechanisms of exosome action are still unknown, and critical questions regarding their potential to trigger immune responses and other safety concerns require further investigation in future experiments. Looking forward, we anticipate that continued research on exosomes will yield novel insights into precision therapy for HCC, thereby improving patient prognosis and quality of life.With sustained efforts, exosomes are poised to emerge as a significant tool in the diagnosis and treatment of HCC, offering patients more effective therapeutic options.
